# Advanced lung adenocarcinomas with *ROS1*-rearrangement frequently show hepatoid cell

**DOI:** 10.18632/oncotarget.12364

**Published:** 2016-09-30

**Authors:** Jing Zhao, Jing Zheng, Mei Kong, Jianya Zhou, Wei Ding, Jianying Zhou

**Affiliations:** ^1^ Department of Pathology, the First Affiliated Hospital, College of Medicine, Zhejiang University, Hangzhou, China; ^2^ Department of Respiratory Disease, Thoracic Disease Center, The First Affiliated Hospital, College of Medicine, Zhejiang University, Hangzhou, China

**Keywords:** NSCLCs, ROS1 rearrangement, clinicopathological features, biopsy

## Abstract

Defining distinctive histologic characteristics of *ROS1*-rearranged non-small-cell lung carcinomas (NSCLCs) may help identify cases that merit molecular testing. However, the majority of previous reports have focused on surgical specimens but only limited studies assessed histomorphology of advanced NSCLCs. In order to identify the clinical and histological characteristics of *ROS1*-rearranged advanced NSCLCs, we examined five hundred sixteen Chinese patients with advanced NSCLCs using *ROS1* fluorescence in situ hybridization and real-time polymerase chain reaction and then analyzed for clinical and pathological features. We performed univariate and multivariate analyses to identify predictive factors associated with *ROS1* rearrangement. 19 tumors were identified with *ROS1* rearrangement (3.7% of adenocarcinomas). 16 *ROS1*+ and 122 *ROS1*- samples with available medical records and enough tumor cells were included for histological analysis. Compared with *ROS1*-negative advanced NSCLCs, *ROS1*-rearranged advanced NSCLCs were associated with a younger age at presentation. *ROS1* rearrangements were not significantly associated with sex, smoking history, drinking history and metastatic sites. The most common histological pattern was solid growth (12/16), followed by acinar (4/16) growth. 66.7% cases with solid growth pattern showed hepatoid cytology (8/12) and 75% cases with acinar growth pattern showed a cribriform structure (3/4). 18.8% cases were found to have abundant extracellular mucus or signet-ring cells (3/16). Only one case with solid growth pattern showed psammomatous calcifications. In conclusion, age, hepatoid cytology and cribriform structure are the independent predictors for *ROS1*-rearranged advanced NSCLCs, recognizing these may be helpful in finding candidates for genomic alterations, especially when available tissue samples are limited.

## INTRODUCTION

Lung carcinomas are the leading cause of cancer-related mortality worldwide [1]. Adenocarcinoma is the most common histologic type of lung cancer [2]. With the development of targeted therapy, adenocarcinoma are classified into subsets, such as epidermal growth factor receptor (*EGFR*)-mutated type [3, 4], anaplastic lymphoma kinase (*ALK*)-rearranged type [5] and *ROS1*-rearranged type [6]. The *ROS1-*rearranged non-small-cell lung carcinomas (NSCLCs) have been reported to be sensitive to the kinase inhibitor crizotinib [7].

Correlation between histology and genotype has been described in lung adenocarcinomas. For example, several studies have suggested that *ALK*-rearranged lung adenocarcinomas were most likely to be observed in the cases with signet-ring cells [8–11]. Boland et al found that 26% pulmonary adenocarcinoma with signet ring cell features were *ALK* rearrangement [8]. However, comprehensive clinical and histological characteristics of *ROS1* rearrangement were not well known given the rarity of this subtype. Data describing the morphologic features of *ROS1*-rearranged lung adenocarcinomas are primarily based on analysis of resected primary lung tumors [12–14]. However, approximately 70% of patients are at advanced stage when diagnosed. For these patients, the small biopsies or cytological specimens are the only limited pathologic material available for diagnosis and molecular testing. It is still unclear whether the findings from prior studies are applicable to specimens from metastatic lung tumors, small biopsies or cytology specimens.

In this study, we confirmed 19 advanced patients with *ROS1* rearrangement by real-time PCR and ROS1 FISH and compared their clinical features and histological characteristics with *ROS1*-subtype. Furthermore, we identified independent predictors for *ROS1* rearrangement by multiple logistic regression analysis.

## RESULTS

### Clinical Characteristics of patients with *ROS1*-rearranged NSCLCs

Of the 516 consecutive patients from the advanced NSCLCs, *ROS1* rearrangements were detected in 19 cases (3.7%) using real-time PCR, 488 cases were negative and 9 cases failed. The tissues were available for FISH in 513 of 516 cases. One lymph node biopsy and two biopsies can't be detected by FISH because of few tumor cells and twelve cases were not interpretable. 18 cases were confirmed positive in both real-time PCR and ROS1 FISH. One lymph node biopsy was eliminated because of few tumor cells. In ROS1 FISH positive patients (n=18), a split pattern was observed in 17 tumors (94.4%), and an isolated red pattern was observed in one tumors (5.6%). One lymph node biopsy with few tumor cells and two cell blocks were not included in histological assessment. The clinical features of 16 patients with *ROS1*-rearranged were summarized in Table 1. *ROS1*-rearranged advanced NSCLCs were associated with a younger age at presentation. Patients with *ROS1*-rearranged tumors had an age range of 38–67 years and mean age of 50 years (compared with mean age of 59 years for patients with *ROS1*-wildtype tumors, *P*=0.001). *ROS1* rearrangements were not significantly associated with sex, smoking history, drinking history and metastatic sites (Table 2).

**Table 1:** Clinical characteristics of patients tested in our study

		N=516	
Mean age (range)		58	(25-79)
Sex	Male	269	
	Female	247	
Smoking status			
	never	287	
	Former	229	
Gene status			
	*EGFR* mutated	241	
	*ALK* rearranged	26	
	Pan WT	230	
	*ROS1* rearranged	19	

**Table 2:** Clinicopathologic features of *ROS1*+ and *ROS1*- advanced lung adenocarcinomas

		*ROS1*+(N=16)	*ROS1*−(N=122)	*P*-value
Age		50	59	0.001*
Sex(M:F)		6:10	66:56	0.328
Smoking status	0	10	67	0.317
	<20	2	8	
	≥20	4	47	
Drinking status	NO	11	91	0.784
	Yes	5	31	
Metastatic sites	Bone	5	57	0.370
	Brain	2	27	0.333
	Adrenal	3	17	0.629
	Liver	1	17	0.323
	Lung	3	38	0.588
	Extra thoracic lymph nodes	4	23	0.151
Histomorphology				
Any Lepidic pattern		0	6	1.000
Any Acinar pattern		4	49	0.286
Any Papillary pattern		0	9	0.598
Any Solid pattern		12	76	0.417
Any Micropapillary pattern		0	4	1.000
Cribriform feature		3	4	0.034*
Extracellular mucus		1	3	0.393
Signet-ring cells		2	5	0.187
Psammoma body		1	4	0.465
Hepatiod cell		8	10	0.016*
Cytological atypia	mild atypia	0	28	0035*
	moderate atypia	8	63	
	severe atypia	8	31	

M: male; F: female.

* indicates parameters showing statistical significance.

### Histologic Characteristics of *ROS1*-rearranged NSCLCs

**Figure 1:**
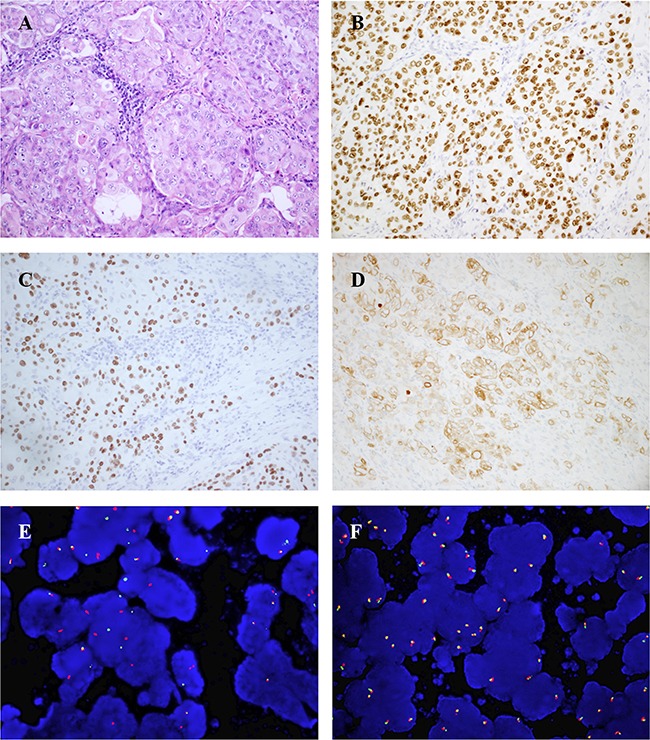
Representative IHC and FISH results of *ROS1*-rearranged advanced lung tumors (case9) **(A)**, solid-predominant growth pattern with hepatoid tumor cells in a metastatic lung adenocarcinoma (hematoxylin and eosin, ×200). **(B-D)**, Tumor cells diffusely co-expressed TTF-1 (B, TTF-1 immunostain, ×200), p40 (C, p40 immunostain, ×200) and CK5/6(D, CK5/6 immunostain, ×200). **(E)**, *ROS1* FISH result using break-apart probes showed *ROS1* rearrangement (splitting of green 5′ and orange 3′ signals). **(F)**, *ROS1* wild type.

**Figure 2:**
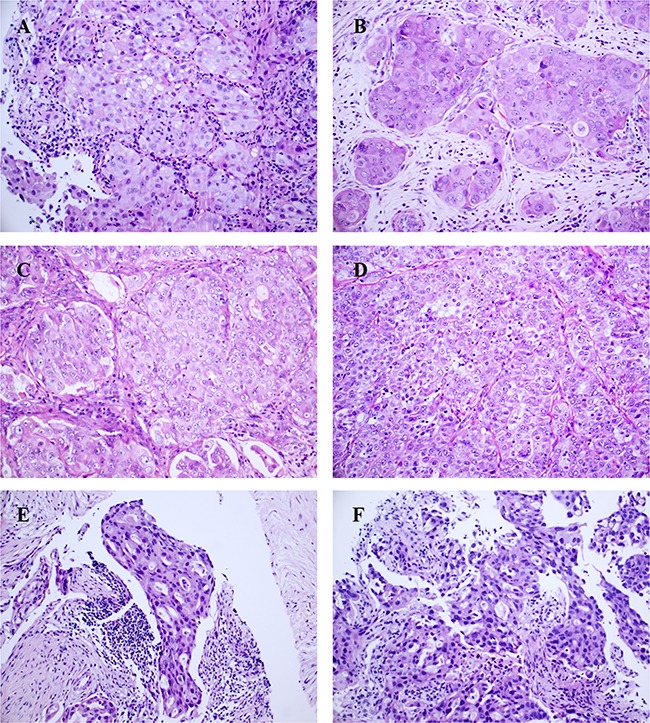
Representative growth patterns of ROS1-rearranged advanced lung tumors (hematoxylin and eosin, ×200): The solid-predominant growth pattern with hepatoid tumor cells in a primary lung tumor **(A)** and 3 lymph node metastasis **(B-D)**, hepatoid tumor cell shows abundant eosinophilic cytoplasm, round nuclei and prominent nucleoli; the acinar-predominant growth pattern with cribriform features in a lymph node metastasis **(E)** and a primary lung tumor **(F)**.

All 16 *ROS1*-rearranged tumors were adenocarcinomas (Supplementary Table S1). In 5 tumors, diffuse co-expression of TTF-1 and p63 was observed, and 2 of them were also positive for CK5/6, and 4 of them were positive for p40 (Figure 1). The histologic characteristics of *ROS1*-rearranged NSCLCs were described in detail in Table 2. The solid growth pattern was the most commonly observed in 75% (12/16) of *ROS1*-rearranged tumors, followed by the acinar-pattern (25%, 4/16) (Figure 2). None of 16 cases was lepidic, papillary or micropapillary growth pattern. 66.7% cases with solid growth pattern showed hepatoid cytology (8/12) (Figure 2A–2D), which had eosinophilic cytoplasm, round nuclei and obvious nucleoli. These 8 cases were negative for antibodies Hep, AFP and Gpc3, and serum alpha-fetoprotein (AFP) were also low (0.6-2.8ng/ml). 75% cases with acinar growth pattern showed a cribriform structure (3/4) (Figure 2E–2F). 18.8% cases were found to have abundant extracellular mucus or signet-ring cells (3/16). Only one case with solid growth pattern showed psammomatous calcifications.

### Comparison between *ROS1*-positive and *ROS1*-negative cases

Clinical and histologic features of 16 *ROS1*-rearranged NSCLCs were compared with those of 122 *ROS1*-negative NSCLCs (Table 2). Age (P=0.001), cribriform feature (P=0.034), hepatoid cytology (P=0.016) and cytological atypia (P=0.035) were associated with *ROS1*- rearranged NSCLCs.

### Multiple Logistic Regression Analysis

We performed multiple logistic regression analysis to identify the independent predictors for *ROS1* rearrangement status. The parameters (age, hepatoid cytology, cribriform structure and cytological atypia, P < 0.1) were included in multivariate analysis. Age, hepatoid cytology and cribriform structure were identified as independent predictors for *ROS1* rearrangement. The significant prediction factors for *ROS1* rearrangement were shown in Table 3.

**Table 3:** The result of multiple logistic regression analysis

Variables	β-Coefficient	Wald Test	*p*-Value	OR	95%CI
Cribriform feature	1.864	3.967	0.046	6.453	1.030-40.417
Hepatiod cell	1.639	5.324	0.021	5.149	1.28-20.718
Age	−0.063	5.391	0.020	0.939	0.890-0.990
Constant	0.021	0.000	0.991	1.021	

## DISCUSSION

Our analysis of 138 advanced NSCLC cases with available clinical data and enough tissue identified age, hepatoid cytology and cribriform structure as the independent predictors for *ROS1* rearrangement, which may help to find candidates for genomic alterations, especially when available tissue samples are limited.

Our study revealed that 3.7% (19 out of 516) of advanced lung adenocarcinomas harbored *ROS1* rearrangement. This prevalence was a little higher than that of the entire adenocarcinoma cohort. This may due to all our patients were in IV stage. Most literature reported *ROS1* rearrangement in 1-2% adenocarcinoma lung, but more than 3% in IV-stage lung adenocarcinoma. Bergethon et al screened 1,073 patients with NSCLC, 18 (1.7%) were *ROS1*-rearranged by FISH [15]. In IV-stage lung adenocarcinoma, 3.4% (11/327) were *ROS1*-rearranged. Kim et al found 3 (3.5%) of 86 IV-stage lung adenocarcinomas harbored *ROS1* rearrangements [16]. What's more, 3 cases in 98 IV-stage lung adenocarcinomas (3/98, 3.1%) were *ROS1* rearrangement in the cohort of Sholl et al [17]. Many reports also found that *ROS1* rearrangement was enriched in advanced group. Scheffler et al found 19 patients of 1035 (1.8%) had *ROS1* rearrangement [18]. The majority of patients presented with stage IV disease at diagnosis (73.7%, 14/19). Mazieres et al identified 32 patients with ROS1 FISH-positive lung cancer in 16 centers from six European countries, and 80.7% (25/32) of these patients were IV-stage lung adenocarcinoma [19].

A correlation between histomorphology and *ROS1*-rearranged lung adenocarcinomas has been demonstrated in prior reports. The majority of the reports have focused on surgical specimens, but only limited studies assessed histomorphology of biopsies, which are procured frequently, especially from patients presenting at an advanced stage of disease and for whom crizotinib is recommended. To the best of our knowledge this is the largest series of *ROS1*-rearranged advanced cases studied to clarify the clinicopathological features. We found that *ROS1*-rearranged advanced lung adenocarcinomas were characterized by two growth patterns: solid growth (12/16) and acinar (4/16) growth. A lepidic pattern was absent in our series, which was similar with other studies [13, 20]. Noguchi suggested that lung adenocarcinoma develop from atypical adenomatous hyperplasia (AAH) through lepidic pattern, and finally invasive adenocarcinoma, being the solid pattern the less differentiated adenocarcinoma component [21]. Infrequent existence with lepidic pattern in *ROS1*-rearranged NSCLCs may lead to the hypothesis that *ROS1* may be a strong oncogenic driver in NSCLCs, and the oncogenic drive of the *ROS1* fusion gene might be powerful enough to bypass the lepidic phase.

The majority of *ROS1*-rearranged advanced lung adenocarcinomas in our series showed a solid growth pattern (75%, 12/16). Interestingly, solid pattern often possessed hepatoid cell (66.7%, 8/12), which showed abundant eosinophilic cytoplasm, round nuclei, and prominent nucleoli. This finding was in agreement with previous reports [22]. Khozin et al reported a patient with hepatoid carcinoma of the lung harbored *ALK* gene rearrangement [23]. Hepatoid adenocarcinomas have been defined as AFP-producing adenocarcinomas with morphologic features similar to hepatocellular carcinoma [24–26]. But 8 cases in our study were negative for antibodies Hep, AFP and Gpc3, and serum alpha-fetoprotein (AFP) were also low (0.6-2.8ng/ml). These finding suggested that these 8 cases with hepatoid cell were not hepatoid adenocarcinomas. We defined them as “poorly differentiated adenocarcinomas with solid growth pattern and eosinophilic cytoplasm”. Careful attention to this feature may lead to find the candidate of advanced NSCLC cases with *ROS1* rearrangement.

Immunohistochemically, 5 of our cases co-expressed TTF-1 and p63, and 4 of them were positive for p40, and 2 cases were also positive for CK5/6. P40, an isoform of p63, has been proposed to be a specific marker of squamous cell carcinoma. In contrast with p63, the expression of p40 was more specific for squamous cell carcinoma [27]. Recent studies reported that 4 cases exhibited unequivocal squamous differentiation, co-expression for TTF1 and p63 [12, 20, 28]. Yonshida et al suggested TTF-1/p63 co-expression occurred frequently in *ALK*-positive NSCLCs [29]. But actually Rekhtman et al found that only 5.4% lung adenocarcinomas co-express TTF-1and p63 in general [30], even less for TTF1 and p40 co-expression. Zhao et al found that *ALK*, *ROS1* or *RET* gene rearrangements appeared 0 times in 214 cases of lung squamous cancer [31], suggesting that the prevalence of *ROS1* rearrangement is very low in SCC histology. We suppose that histology of *ROS1*+ lung cancer has low differentiation with peculiar immunoprofile, which may indicate that the cell of origin of *ROS1*+ lung adenocarcinoma may different from most lung adenocarcinomas.

In our series, signet-ring cells presented only in two cases (2/12), which seems lower in previous reports with surgical cases (33-55%) [12, 20], and much lower than *ALK*-rearranged lung adenocarcinoma (71%) [11]. Furthermore, only one case with solid growth pattern showed psammomatous calcifications, while Sholl et al found psammomatous calcifications were highly associated with *ROS1* rearrangement (6 of 9 or 66%vs. 1 of 185 or 0.5%; P<0.001) as compared with *ROS1* wild type [17]. These differences may be due to the differences in specimen source (lung resection vs. biopsy) and tumor stage (early vs. advanced). Some features frequently presented in lung resections may be missed because of the limited tissue samples. Thus, it is essential to find out the features of advanced lung adenocarcinoma with *ROS1* rearrangement.

The results of our study highlight that hepatoid cytology and cribriform structure may be the key morphologic features associated with advanced lung adenocarcinomas with *ROS1* rearrangement. The recognition of hepatoid cytology and cribriform structure could help to triage a specimen for appropriate molecular testing, especially when tissue is limited. However, the diagnosis of *ROS1*-rearrangement needs to be validated by FISH or real-time PCR.

## MATERIALS AND METHODS

### Patients and samples

The study was approved by the Ethics Committee of the First Affiliated Hospital of Zhejiang University. The Ethics Committee waived the need for consent for the use of the samples in this research study. Our study group consisted of 516 consecutive cases of IV-stage lung adenocarcinoma diagnosed at the First Affiliated Hospital of Zhejiang University between 2014 and 2016, 19 of which were *ROS1*+ and 497 of which were *ROS1*-. *ROS1* rearrangements were detected using real-time PCR and FISH, as described below. The specimen types consisted of small biopsies (n=499) and cell blocks (n=13) or Ebus (n=4). The clinical characteristics of all patients are summarized in Table 1. In order to clarify clinicopathological characteristics of *ROS1* rearrangement, samples with available medical records and enough tumor cells for histological analysis were separated into two groups: 16 *ROS1*+ and 122 *ROS1*-. 3 *ROS1*-rearranged cases (1 biopsy and 2 cell blocks) have too few tumor cells to histological analysis.

### Pathology review

Hematoxylin and eosin (H&E)-stained slides of all samples were reviewed by two experienced pathologists (MK and WD) to evaluate the histologic characteristics based on the 2015 WHO classification of lung adenocarcinoma [32]. Immunohistochemistry analyses for P63 (clone 7JUL, Long island bio, Shanghai, China), P40 (cloneBC28, ZSGB-BIO, Beijing, China), CK5/6 (clone D5/16B4, Long island bio, Shanghai, China), CK7 (clone OV-TL12/30, Long island bio, Shanghai, China), and TTF-1 (clone SPT24, ZSGB-BIO, Beijing, China) were performed to differentiate between adenocarcinoma and squamous cell carcinoma. Antibodies for Hep (Hepatocyte, clone OCH1E5, ZSGB-BIO, Beijing, China), AFP (clone ZSA06, ZSGB-BIO, Beijing, China) and Gpc3 (Glypican-3, clone 1G12, ZSGB-BIO, Beijing, China) were evaluated in 5 tumors with hepatoid cytology. Immunohistochemistry was performed on 4 μm-thick FFPE tissues using Ventana automated immunostainer (Ventana Medical Systems, Tucson, AZ) according to the manufacturer's protocol. Antigen retrieving was performed with EDTA (pH9.0) for 20 minutes. Cytological atypia of tumors were classified as mild atypia (relatively uniform nuclei with indistinct nucleoli at 100 × magnification), moderate atypia (relatively uniform nuclei with distinct nucleoli at 100 × magnifications) and severe atypia (bizarre, enlarged nuclei of varied sizes, with some nuclei at least twice as large as others) [33]. The highest degree of atypia was recorded for heterogeneous tumors. Hepatoid cytology was defined as the solid pattern with hepatoid tumor appearance characterized by abundant eosinophilic cytoplasm, round and relatively monomorphic nuclei, and prominent nucleoli [11].

### Fluorescence *In Situ* hybridization

Slices of FFPE tissues, 4μm thick, were used to evaluate the presence of *ROS1* gene fusion by FISH, using a break-apart probe for ROS1 (6q22 ROS1 Break Apart FISH Probe; Abbott Molecular, Des Plaines, IL, USA). Nuclei were counterstained with DAPI (Zytovision, Bremerhaven, Germany) and slides were examined with a BX51 fluorescence microscope (Olympus, Tokyo, Japan) equipped with a triple-pass filter (DAPI/Green/Orange). Samples were considered to be *ROS1* positive if more than 15% of tumor cells showed split red and green signals (signals separated by ≥1 signal diameter) or single 3′ signals. Otherwise the samples were considered as FISH negative.

### Real-time PCR

*ROS1* was amplified by multiplex real-time PCR using a Stratagene Mx3000P real-time PCR system (Stratagene, CA) with an AmoyDx® ROS1 fusion gene detection kit (Amoy Diagnostics Co., Ltd, Xiamen, China). All types of *ROS1* gene fusions detected with the detection kit were listed in Supplementary Table S2. A case was considered positive for *ROS1* rearrangement if FAM Ct value was <30. Otherwise the samples were considered as negative. The sample may contain two or more fusion patterns simultaneously. The status of the EGFR mutation and ALK rearrangement were also analyzed by real-time PCR and IHC, according to methods previously described [34].

### Statistical analysis

Statistical analysis was performed using SPSS software version 17.0 (SPSS Inc., Chicago, IL). Continuous variables were analyzed using Student T test. The categorized variables were analyzed using Chi-square test. Multivariate analysis was performed to determine the discriminating power of each histologic parameter between *ROS1*-positive and *ROS1*-negative cohorts by multiple logistic regressions. P<0.05 was considered statistically significant.

## SUPPLEMENTARY MATERIALS TABLES




