# Supplementary material for: Advanced lung adenocarcinomas with ROS1-rearrangement frequently show hepatoid cell
Source: Oncotarget. 2016 Sep 30;7(45):74162–70. doi: 10.18632/oncotarget.12364 (PMC5342043; doi:10.18632/oncotarget.12364)
Supplement: Supplementary file 1 [file oncotarget-07-74162-s001.pdf]

Advanced lung adenocarcinomas with *ROS1*-rearrangement frequently show hepatoid cell

SUPPLEMENTARY TABLES

Supplementary Table S1: Pathologic Features of ROS1-altered Tumors.

See Supplementary File 1

Supplementary Table S2: *ROS1* gene fusions detected with the detection kit

Tube NO.	Spliced Gene & Exon	ROS1 Spliced exon
1	SLC34A2 exon4, SLC34A2 exon14del, CD74 exon6<br>SDC4 exon2, SDC exon4	32
2	SLC34A2 exon4, SLC34A2 exon14del, CD74 exon6<br>SDC4 exon2, EZR exon10	34
3	TPM3 exon8, LRIG3 exon16, GOPC exon8	35
4	GOPC exon4	36
