# Supplementary material for: Advanced lung adenocarcinomas with ROS1-rearrangement frequently show hepatoid cell
Source: Oncotarget. 2016 Sep 30;7(45):74162–70. doi: 10.18632/oncotarget.12364 (PMC5342043; doi:10.18632/oncotarget.12364)
Supplement: Supplementary file 2 [file oncotarget-07-74162-s002.doc]

**Table S1. Pathologic Features of *ROS1*-altered Tumors**

|  | Sex | Age | Specimen Type | Smoking (pack-years) | Predominant Growth Pattern | Signet-ring Cells | Extracellular Mucus | Hepatiod Cell | Cytologic Atypia | Cribriform Feature | Psammoma Body | CK5/6 | P40 |
| --- | --- | --- | --- | --- | --- | --- | --- | --- | --- | --- | --- | --- | --- |
| 1 | M | 42 | Transthoracic lung core biopsy | 20 | Solid | 0 | 0 | 1 | moderate | 0 | 0 | － | focal ＋ |
| 2 | F | 49 | Transthoracic lung core biopsy | 0 | Solid | 0 | 0 | 1 | moderate | 0 | 0 | － | － |
| 3 | F | 67 | Bronchoscopic biopsy | 0 | Solid | 0 | 0 | 0 | severe | 0 | 0 | － | － |
| 4 | F | 45 | Transthoracic lung core biopsy | 0 | Solid | 0 | 0 | 1 | severe | 0 | 0 | － | － |
| 5 | M | 57 | Lymph node biopsy | 40 | Solid | 0 | 0 | 0 | moderate | 0 | 0 | － | － |
| 6 | M | 56 | Transthoracic lung core biopsy | 7.5 | Solid | 1 | 0 | 0 | moderate | 0 | 0 | ＋ | ＋ |
| 7 | F | 46 | Transthoracic lung core biopsy | 0 | Solid | 1 | 0 | 0 | moderate | 0 | 0 | － | － |
| 8 | M | 60 | Lymph node biopsy | 54 | Solid | 0 | 0 | 1 | severe | 0 | 0 | － | － |
| 9 | F | 41 | Lymph node biopsy | 0 | Solid | 0 | 0 | 1 | severe | 0 | 1 | ＋ | ＋ |
| 10 | F | 58 | Bronchoscopic biopsy | 0 | Solid | 0 | 0 | 1 | severe | 0 | 0 | － | － |
| 11 | F | 45 | Lymph node biopsy | 0 | Solid | 0 | 0 | 1 | moderate | 0 | 0 | － | － |
| 12 | F | 38 | Transthoracic lung core biopsy | 0 | Solid | 0 | 0 | 1 | severe | 0 | 0 | － | － |
| 13 | M | 50 | Transthoracic lung core biopsy | 30 | Acinar | 0 | 0 | 0 | moderate | 1 | 0 | － | focal ＋ |
| 14 | F | 62 | Pleural biopsy | 0 | Acinar | 0 | 0 | 0 | severe | 0 | 0 | － | － |
| 15 | M | 52 | Lymph node biopsy | 1 | Acinar | 0 | 0 | 0 | severe | 1 | 0 | － | － |
| 16 | F | 39 | Transthoracic lung core biopsy | 0 | Acinar | 0 | 1 | 0 | moderate | 1 | 0 | － | － |
